# CACNA1H downregulation induces skeletal muscle atrophy involving endoplasmic reticulum stress activation and autophagy flux blockade

**DOI:** 10.1038/s41419-020-2484-2

**Published:** 2020-04-24

**Authors:** Suting Li, Menglei Hao, Bingshu Li, Mao Chen, Jue Chen, Jianming Tang, Shasha Hong, Jie Min, Ming Hu, Li Hong

**Affiliations:** Department of Gynecology and Obstetrics, Renmin Hospital of Wuhan University, Wuhan, Hubei Province People’s Republic of China

**Keywords:** Molecular biology, Pathogenesis

## Abstract

Multiple vaginal delivery (MVD) is an important factor for pelvic floor muscle (PFM) function decline and pelvic floor dysfunction (PFD). PFD is common in middle-aged and elderly women, but its pathogenesis is not clear. In this study, we found that the expression of CACNA1H was lower in the PFM of old mice after MVD compared with old or adult mice. In in-vitro studies, we found that treatment with the T-type Ca^2+^ channel (T-channel) inhibitor NNC-55 or downregulation of the CACNA1H gene by siRNA intervention promoted myotube atrophy and apoptosis. Mechanistically, we revealed that NNC-55 increased the expression of GRP78 and DDIT3 in myotubes, indicating endoplasmic reticulum stress (ERS) activation, and that the IRE1 and PERK pathways might be involved in this effect. NNC-55 induced the formation of autophagosomes but inhibited autophagy flux. Moreover, rapamycin, an autophagy activator, did not rescue myotube atrophy or apoptosis induced by NNC-55, and the autophagy inhibitors 3-MA and HCQ accelerated this damage. Further studies showed that the ERS inhibitors 4-PBA and TUDAC relieved NNC-55-induced damage and autophagy flux blockade. Finally, we found multisite muscle atrophy and decreased muscle function in Cacna1h^−/−^ (TH-null) mice, as well as increased autophagy inhibition and apoptotic signals in the PFM of old WT mice after MVD and TH-null mice. Taken together, our results suggest that MVD-associated PFD is partially attributed to CACNA1H downregulation-induced PFM atrophy and that ERS is a potential therapeutic target for this disease.

## Introduction

Skeletal muscle is one of the most important organs in the body, comprising approximate 50% of the total body weight. The pelvic floor muscle (PFM) is an essential constituent of the pelvic floor, and its dysfunction contributes to the pathogenesis of pelvic floor dysfunction (PFD), including pelvic organ prolapse and urinary and fecal incontinence. These morbid conditions affect women around the world, especially older parous women and are accompanied by a significant economic burden on patients and the health care system^1–3^. Thus, substantial efforts have been made to study the pathogenesis and treatment of these diseases^4^. Age- and multiple vaginal delivery (MVD)-related detrimental alterations in muscle architecture, the primary determinant of muscle function, have been shown to occur in the PFM^5,6^. Although the muscle mass and sarcomere length in older individuals did not differ significantly from those in younger individuals, the force-producing capacity, as assessed by physiological cross-sectional area, was substantially lower in older individuals than in younger individuals^7^.

T-type Ca^2+^ channels (T-channels) can be activated by small depolarizations of the plasma membrane and have been implicated in a variety of physiological processes, including fertilization, neuronal firing, cancer therapy, hormone secretion and muscle fiber development^8–12^. Bijlenga^13^. showed that T-channels were expressed in myoblasts just before fusion, which plays an important role in Ca^2+^ flow in terminally differentiated myoblasts. There are three types of T-channels with different α subunits: Cav3.1, Cav3.2, and Cav3.3, which are encoded by CACNA1G, CACNA1H and CACNA1I, respectively. Among these three types of T-channels, the expression of CACNA1H in mammalian skeletal muscle and myoblasts is higher than that of the other two types, as shown in our study and those of others^12,13^. Previous studies have shown an age-related decline in the T-type Ca^2+^ current in skeletal muscle^14^. It was also recently reported that the compound heterozygous mutation of CACNA1H led to congenital muscular atrophy, perhaps through dysfunctional Ca^2+^ signaling^15^.

Skeletal muscle contains a large amount of endoplasmic reticulum (ER), called the sarcoplasmic reticulum (SR), which plays an important role in regulating the internal stability of skeletal muscle. Endoplasmic reticulum stress (ERS) can be caused by an imbalance of Ca^2+^, excess reactive oxygen species, the accumulation of misfolded proteins, and many other perturbations that disrupt cell homeostasis^16–18^. There are three ER-resident proximal sensors of unfolded proteins and ERS: ATF6, IRE1, and PERK^19,20^. Normally, GRP78 can bind these three molecules at the inner end of the ER to maintain their resting state. Under traumatic stimulation, GRP78 expression increases to reduce or stop the ERS response, restoring intracellular homeostasis, but chronic, unmitigated ERS can lead to the activation of ERS-related proapoptotic factors and finally promote the occurrence of apoptosis^21^. Although the role of ERS in the maintenance of skeletal muscle function is controversial, several studies have shown that chronic, long-term ERS can affect and impair muscle function in a variety of ways^22–24^.

Autophagy is a self-digestive cellular stress response that enables cells to promote survival^25^. Macroautophagy, which is generally characterized by initial autophagosome formation followed by autophagosome-lysosome fusion and ultimately lysosomal enzyme degradation to achieve cell homeostasis and organelle renewal, is the main type of autophagy^26^. The blockade of any of the above steps leads to a decline in autophagy flux. Autophagy activation can protect cells from stimulated injury by removing dysfunctional organelles or excessive misfolded proteins^27^. Decreased autophagy is one of the causes of age-related muscular atrophy, and the activation of autophagy can protect muscles and maintain their normal functions^28^. Studies have shown that autophagy is regulated by ERS^29,30^, but the regulatory mechanisms are ambiguous. In the study of tumor cells, T-channel inhibitors or downregulation of T-channel subtypes was found to regulate the apoptosis response, and the relevant mechanisms involved ERS activation and autophagy inhibition^31–33^. However, T-channel function in skeletal muscle has been poorly studied.

Here, we report that CACNA1H expression in the PFM of old mice after MVD was decreased and that inhibition of T-channels could induce ERS and block autophagy flux in myotubes. In addition, ERS inhibition is an important factor in protecting myotubes from T-channel inhibitor-induced atrophy and apoptosis. These findings have broad implications for understanding the genes and pathways that regulate PFM function and provide insights into the role of CACNA1H as a critical regulator of skeletal muscle function.

## Materials and methods

### Mice and Study Design

Mouse breeding and experimentation were performed in compliance with the institutional guidelines and regulations of Wuhan University. All of the procedures that involved animals were approved by the Institutional Animal Care and Use Committee of Renmin Hospital of Wuhan University (license number: WDRM 2016-1105). We purchased wild-type female C57BL/6J mice from the Center for Animal Experiment of Wuhan University and Cacna1h^−/−^ mice (TH-null) from the Jackson Laboratory (Stock 013770; Bar Harbor, ME, USA). The mice of the two types were randomly divided into four groups (*n* = 7) for feeding and experiment in our study: the youth group (1-month old), the adult group (virgin, 4-month old), aged group (virgin, 12-month old) and aged multiple vaginal-delivery (Aged MVD, 6-8 times of gestation and vaginal-delivery and no lactation, 12-month old) group. The specific procedures of PFM collection could be seen in our previous study^34^. Mice were housed in the Animal Experimental Center and Institute of Model Animal of Wuhan University during these experiments. Details of the identification and validation of Cacna1h^−/−^ mice are provided in Supplementary Fig. 1a.

### C2C12 myoblast culture and differentiation

Immortalized murine C2C12 myoblasts were purchased from Cobioer Biosciences Co., Ltd. (Nanjing, China). Myoblasts were cultured in Dulbecco’s modified Eagle’s medium (DMEM) (4.5 g/L d-glucose) supplemented with 10% fetal bovine serum (FBS) and 1% penicillin–streptomycin in an incubator with 5% CO_2_ at 37 °C. After reaching 80–90% confluence, C2C12 myoblasts were transferred into differentiation medium (DMEM supplemented with 2% horse serum, 1% penicillin–streptomycin) for 4 days following with drug intervention for 1–2 days.

### Chemicals and reagents

NNC 55-0396 hydrate (NNC-55) was purchased from Sigma-Aldrich (N0287). NNC-55 was dissolved in DMSO (Sigma-Aldrich, D2650) and stored at -20°C or -80°C. Other reagent sources are listed below: FBS, horse serum, trypsin/EDTA solution, DMEM (GIBCO-BRL, Gaithersburg, MD, USA); 3-Methyladenine (3-MA), hydroxychloroquine (HCQ), 4-phenylbutyric acid (4-PBA), Tauroursodeoxycholate (TUDCA) and collagenase (MCE, Monmouth Junction, USA); Annexin VPE/7-AAD Apoptosis Detection Kit (BD Biosciences, San Diego, CA, USA); Mitochondrial Permeability Transition Pore (mPTP) Assay Kit (YEASEN, Shanghai, China); Fluo-3AM (Sigma-Aldrich, 39294).

### Gene-Expression Analyses

Total RNA isolated with TRIzol (Takara Bio Inc., Otsu, Japan) according to the manufacturer’s instructions was reverse-transcribed into cDNA using Hifair II 1st Strand cDNA Synthesis Kit (YEASEN, Shanghai, China). Quantitative Real-time PCR was carried out using Hieff qPCR SYBR Green Master Mix (No Rox) (YEASEN, Shanghai, China) and the CFX96 Trademark Real-time PCR detection system (Bio-Rad, California, USA). The expression levels of CACNA1G, CACNA1H, ATF6, IRE1, PERK, CTSB, CTSD, LAMP2, MURF1 and Antrogin1 were examined with the primers (Sangon Biotech, China) listed in Supplementary Table 2.

### Western blotting

The preparation of total protein lysates and western blotting were performed as described previously^35^. The primary antibody information was as follows: anti-CACNA1H (1:1000, ImmunoWay, YT4773), anti-β-actin (1:1000, Proteintech, 20536-1-AP), anti-MyHC (1:300, RND, MAB4470), anti-GRP78 (1:1000, Proteintech, 11587-1-AP), anti-DDIT3 (1:1000, CST, #5554), anti-SQSTM1 (1:1000, MBL, M162-3), anti-Beclin-1(1:1000, Proteintech, 11306-1-AP), anti-LC3 (1:1000, Proteintech, 14600-1-AP), anti-cytochrome C (1:1000, CST, #11940).

### Immunofluorescence staining

Extensor digitorum longus from adult mice were digested in type I collagenase at 37 °C for 2-3 h, and dissociated into single fibers. These single fibers and myotubes fixed with 4% paraformaldehyde for 15 min at 4 °C and then performed as described previously^36^. The primary antibody information was as follows: anti-MyHC (1:50, RND, MAB4470), anti-SQSTM1 (1:150, MBL, M162-3), anti-LC3 (1:100, Proteintech, 14600-1-AP).

### Flow cytometric analysis of apoptosis

Myotubes were collected and detected for apoptosis analysis according to the operating instructions. A flow cytometry—fluorescence-activated cell sorting (FACS) Calibur system (BD, Franklin Lakes, NJ, USA) was used to conduct signal collection, and then analyzed with FlowJo software (BD Biosciences).

### Flow cytometric analysis of mPTP

Myotubes were collected and detected for mPTP analysis. FACS Calibur flow cytometry system and FlowJo software were used in this experiment as mentioned above. CoCl_2_ and Ionomycin were added simultaneously as negative controls in each group.

### Flow cytometric analysis of Ca^2+^ concentration

Myotubes were harvested, incubated with Fluo-3AM (10 μM) for 30 min in the incubator and then performed as described previously^36^. The Geo Mean fluorescence intensity was used to compare differences of Ca^2+^ concentration between groups.

### Autophagy flux analysis

The mRFP-GFP-LC3 adenoviral vectors was purchased from HanBio Technology (Shanghai, China). Myotubes were transfected and then incubated in medium containing NNC-55 (10 μM) or/and the ERS inhibitor for the for indicated time in incubator. Autophagy flux observation and mounting were performed with a laser scanning confocal microscopy (FV1200, Olympus Corp, Tokyo, Japan).

### Transmission electron microscope (TEM) analysis

After treatment with NNC-55 (10 μM) for 24 and 48 h, myotubes were fixed in 4% glutaraldehyde, centrifuge for 5 min × 1000 r and then sent to Ultrapathological Center of Renmin Hospital of Wuhan University for analysis. Samples were observed by TEM (HITACHI HT7700, Tokyo, Japan).

### Transfection assay

siRNA duplex oligonucleotides against mouse CACNA1H and the negative control were transfected into myotubes at a final concentration of 50 nM using riboFECT CP Transfection Kit (RIBOBIO, Guangzhou, China).

### Histology and morphometric analysis

PFM, Tibialis Anterior (TA) and Soleus (SOL) of the mice were isolated, fixed in the muscle fixation fluid (Servicebio, China) and embedded in paraffin. To assess tissue morphology, we stained cross-sections with hematoxylin and eosin (H&E). The sections were examined under microscope (BX63, Olympus Corp, Tokyo, Japan). Images were quantified with Image-Pro-Plus software to measure myofiber cross-sectional area (CSA). The distribution of myofiber CSA was calculated by analyzing 500 myofibers per muscle.

### Muscle force measurements in vivo

Mice were anesthetized with isoflurane and placed on the horizontal stage. Optimal muscle length that allows maximal isometric twitch force was determined by a sequence of twitch contractions at 150 Hz every 1 s with small varying changes to muscle tension and current. After muscle optimization, to obtain specific twitch force, a pre-stimulus and post-stimulus baseline of 200 ms was recorded to establish a baseline recording, and a 0.2 ms pulse width was used. In vivo force measurements were conducted with the PowerLab data acquisition and analysis system (AD Instruments, Australia).

### Statistics

The data are presented as mean ± SD. Student’s t-tests (unpaired two-tailed) were applied for comparisons in two groups, and one-way analysis of variance (ANOVA) was used for multiple comparisons in three or more groups. *P* values < 0.05 were considered significant. Statistical analysis was performed with GraphPad Prism software version 7 (San Diego, CA, USA).

## Result

### MVD reduces CACNA1H expression in the PFM

**Fig. 1:**
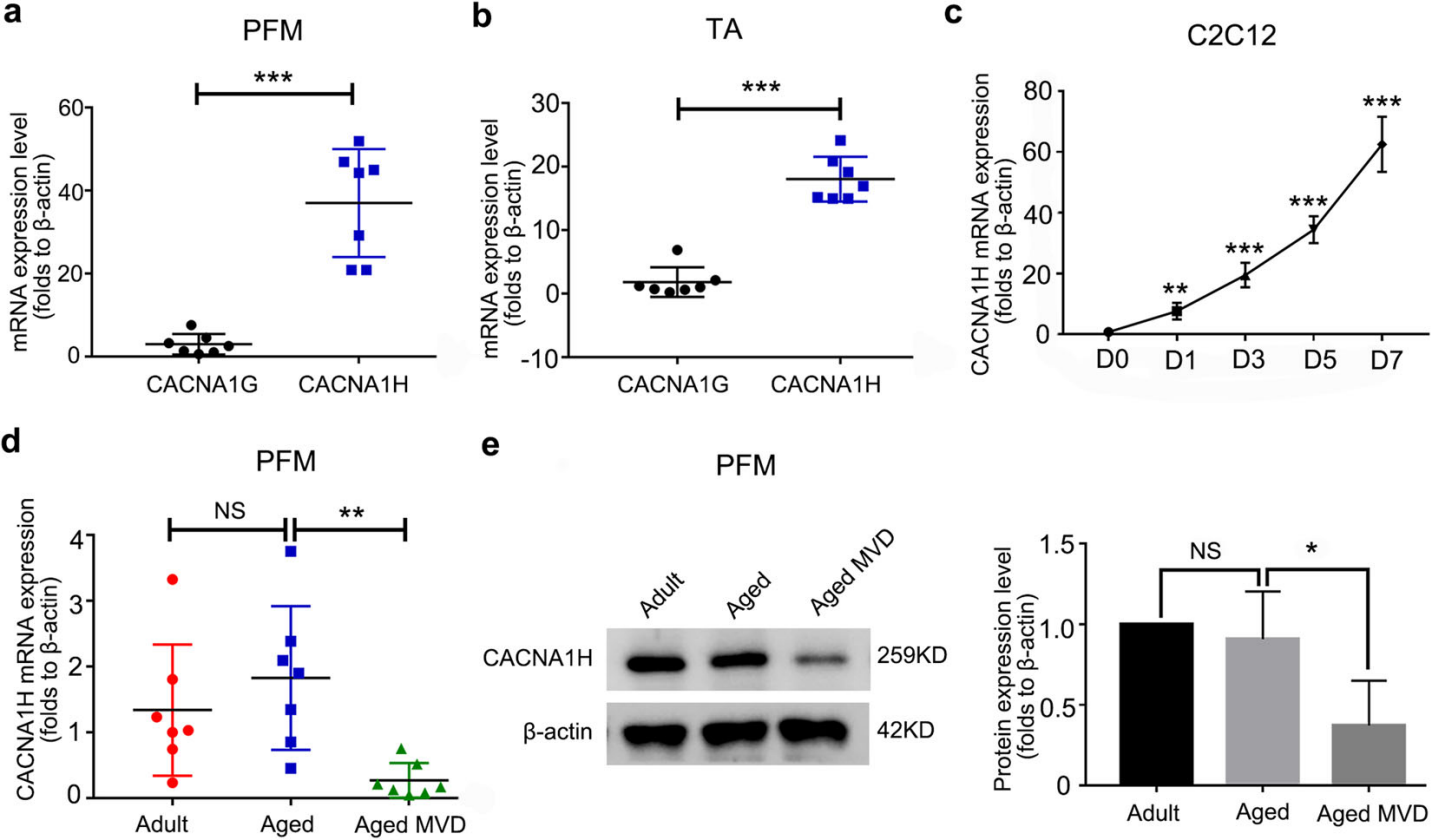
The expression of T-channel in PFM, TA and C2C12 myotubes. **a**, **b** The CACNA1G and CACNA1H mRNA expression were detected in PFM (a) and TA (b) of 4-month-old WT mice (*n* = 7) by qRT-PCR. **c** CACNA1H mRNA expression was examined during the differentiation of C2C12 by qRT-PCR. **d**, **e** The expression of CACNA1H in PFM of the adult virgin (4-month old, *n* = 7), aged virgin (12-month old, *n* = 7) and aged MVD (12-month old, *n* = 7) group mice was analyzed by qRT-PCR (**d**) and western blotting (**e**). These data are presented as (mean ± SD). PFM, pelvic floor muscle. TA, anterior tibial muscle. CON, control. Aged MVD, aged multiple vaginal-delivery. NS, no significance. *P < 0.05, **P < 0.01, ***P < 0.001. **a**, **b** Unpaired two-tailed Student’s *t* test. **c**–**e** One-way analysis of variance (ANOVA).

First, we detected the expression of three type T-channels in the PFM in adult wild-type (WT) mice and found that the mRNA expression of CACNA1H was approximate 12.5 times that of CACNA1G (Fig. 1a). The mRNA expression of CACNA1H in the TA was ~9.9 times to 10 times that of CACNA1G (Fig. 1b).CACNA1H expression was significantly increased during the differentiation of C2C12 cells (Fig. 1c). Furthermore, we detected the expression of CACNA1H in the PFM of adult virgin mice, old virgin mice and old mice after MVD and found that CACNA1H mRNA expression was significantly reduced in the old mice after MVD (Fig. 1d, e). Collectively, our data demonstrate that CACNA1H is the main type of T-channel in the PFM and that pregnancy and MVD can reduce CACNA1H expression in PFM.

### The T-channel inhibitor NNC-55 promotes myotube atrophy, mitochondrial damage and apoptosis

**Fig. 2:**
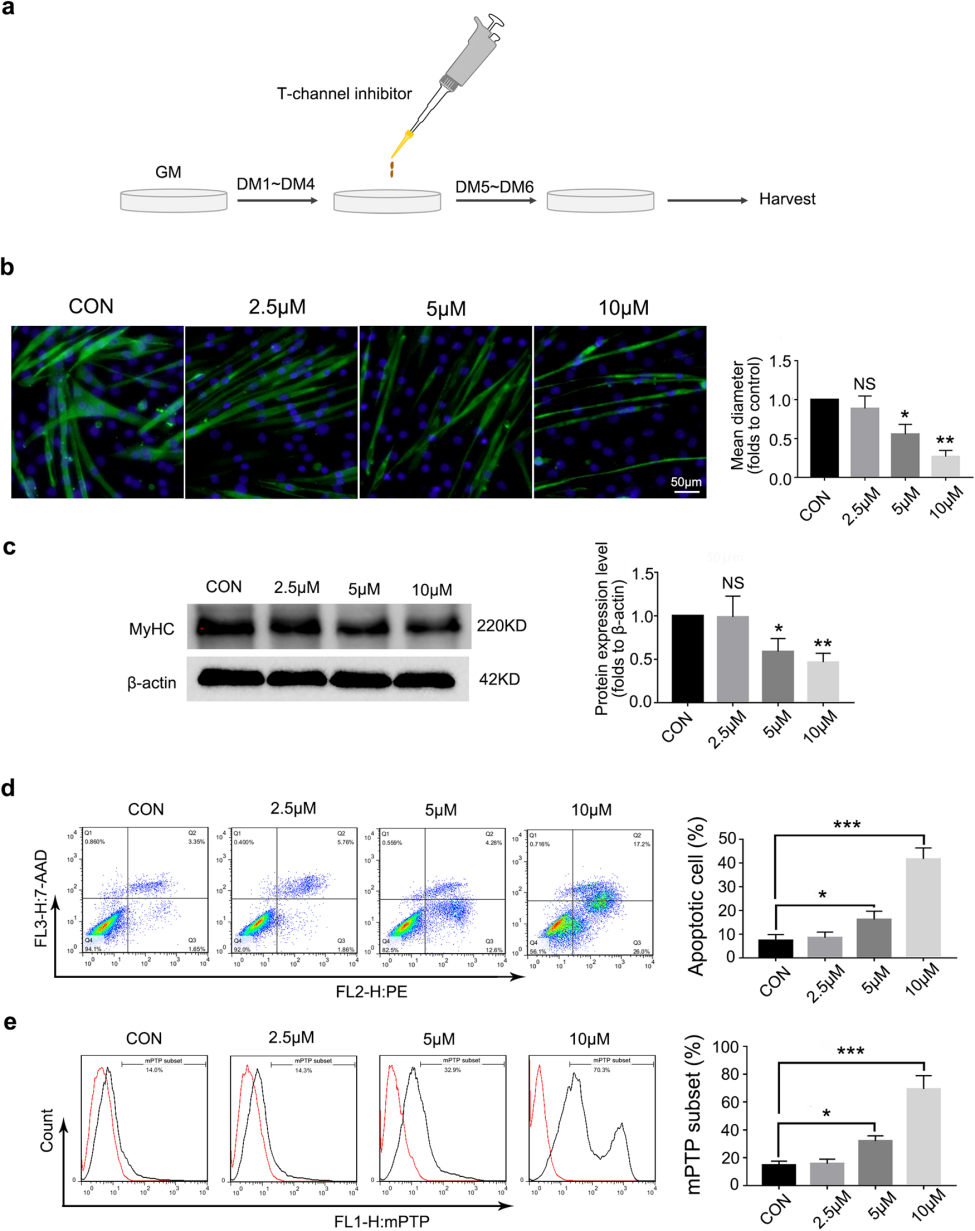
T-channel inhibitor induces myotube atrophy and injury. **a** Schematic diagram illustrates the in vitro experimental process. **b**–**e** Myotubes were incubated with various concentrations (up to 10 μM) of NNC-55 for 48 h, and the myotubes status were analyzed by anti-MyHC immunofluorescence staining (**b**), and western blotting (**c**). Apoptotic myotubes were detected with the annexin V-PE/7-AAD kit (**d**) and mitochondrial permeability were detected by mPTP kit (**e**) and then analyzed by flow cytometry. These data are presented as the (mean ± SD) for three independent experiments. GM, growth medium. DM, differential medium. CON, control. NS, no significance. *P < 0.05, **P < 0.01, ***P < 0.001. One-way analysis of variance (ANOVA).

NNC-55 is a selective T-channel inhibitor^37^. To examine the putative effect of T-channels on muscle atrophy, on day 5 of C2C12 differentiation, we added NNC-55 and incubated the cells for 48 h (Fig. 2a). Our study showed that NNC-55 could reduce myotube diameter in a dose-dependent manner (Fig. 2b), and that the MyHC protein content was also decreased (Fig. 2c). Furthermore, the myotubes apoptosis was detected under treatment with different concentrations of a T-channel inhibitor. The results showed that the apoptosis rate of myotubes increased by almost 3 times compared with that in the control group after 48 h treatment (Fig. 2d). Meanwhile, we detected mitochondrial damage in the myotubes by using mPTP assay kit and found that NNC-55 led to increased damage in a dose-dependent manner (Fig. 2e).

### NNC-55 induces ERS and intracellular Ca^2+^ disorder

**Fig. 3:**
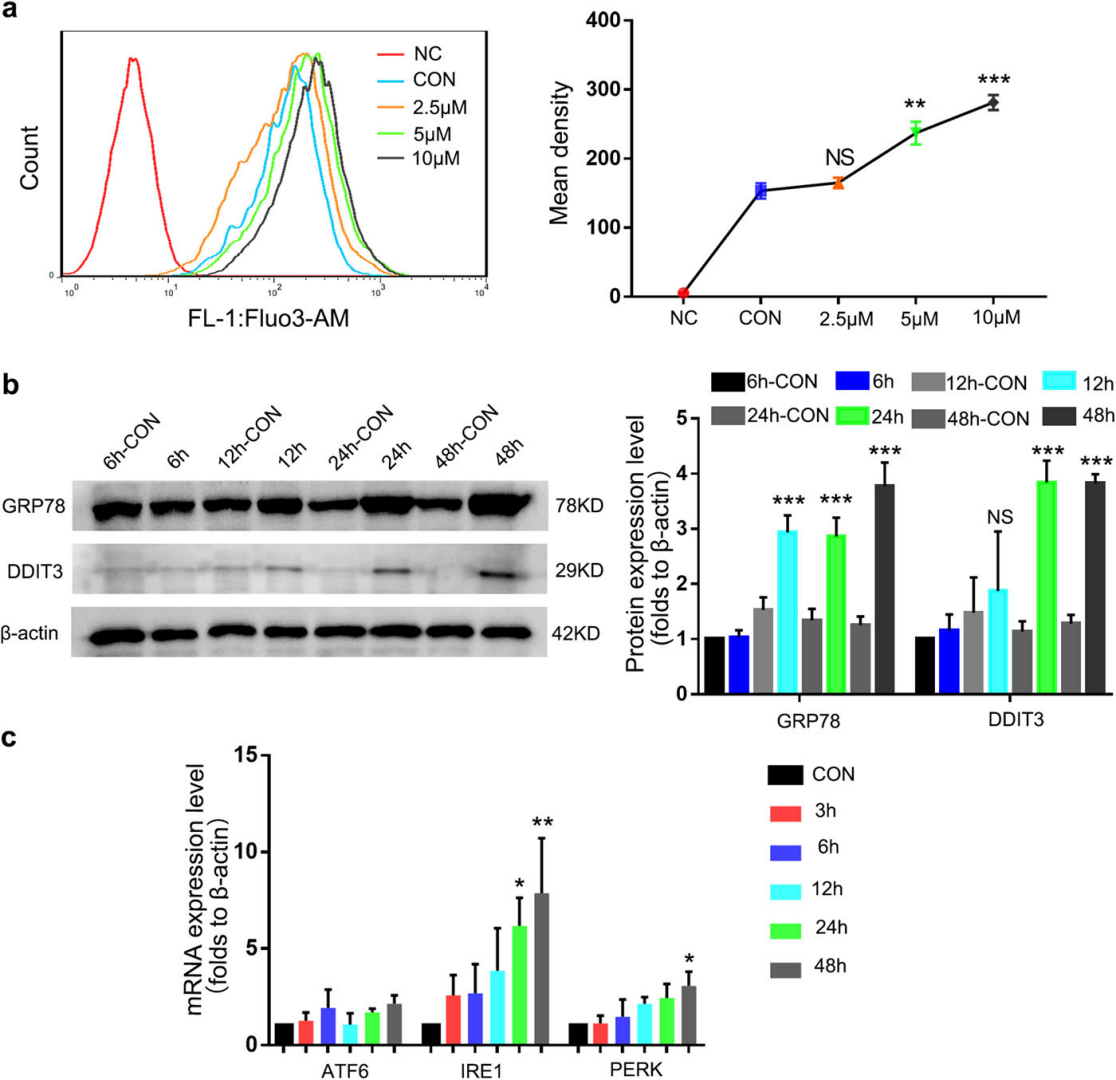
T-channel inhibitor induces ERS and intracellular Ca^2+^ disorder in myotubes. **a** C1C12 myotubes were incubated with various concentrations (up to 10 μM) of NNC-55 for 48 h, and the Ca^2+^ ion concentration in myotubes was indicated by Fluo-3AM and analyzed with flow cytometry. **b**, **c** Myotubes were incubated with NNC-55 (10 μM) for various time (up to 48 h). GRP78, DDIT3 protein expression were analyzed by western blotting (**b**) and ATF6, IRE1 and PERK gene expression were detected by qRT-PCR (**c**). These data are presented as the (mean ± SD) for three independent experiments. NC, negative control. CON, control. NS, no significance. *P < 0.05, **P < 0.01, ***P < 0.001. One-way analysis of variance (ANOVA).

A previous study suggested the ability of T-channels to couple Ca^2+^ influx to ER Ca^2+^ storage^38^, so we asked whether a T-channel inhibitor would disrupt Ca^2+^ homeostasis, ultimately disrupting ER. Initially, we examined the level of Ca^2+^ in myotubes after treatment with different concentrations of NNC-55. The results showed that after T-channel inhibition for 48 h, the Ca^2+^ level in the myotubes increased in a dose-dependent manner, especially under 10 μM (Fig. 3a).

The ER is an important organelle for the regulation of intracellular Ca^2+^ stability, and excessive imbalance in Ca^2+^ homeostasis can eventually lead to ERS^16^. In fact, sustained ERS has been reported to drive muscle atrophy^39^. We thus tested the expression of ERS markers GRP78 and DDIT3. Indeed, we found that the GRP78 and DDIT3 proteins were upregulated in the NNC-55-treated group compared with the control group, and this increase was evident after 24 h of treatment, while there is no difference in the control groups at different time points (Fig. 3b). In addition, we detected important molecules in three ERS signaling pathways, ATF6, IRE1 and PERK and qRT-PCR analysis showed increased expression of IRE1 and PERK (Fig. 3c).

### NNC-55 promotes autophagosome formation but blocks distal autophagy and autophagy flux

**Fig. 4:**
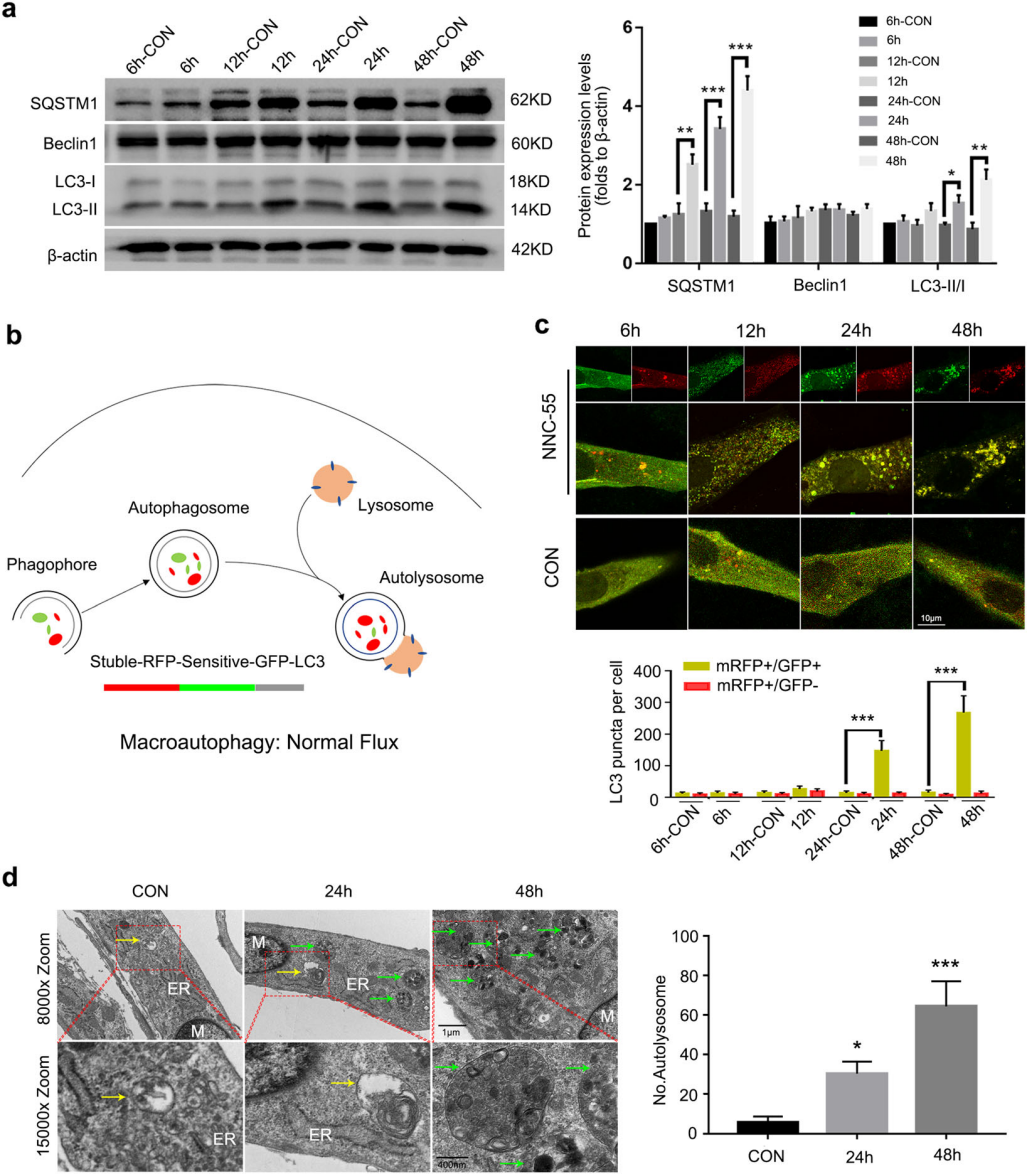
T-channel inhibitor promotes autophagosome formation but blocks autophagy flux in myotubes. **a** Myotubes were incubated with NNC-55 (10 μM) for various time (up to 48 h) and the expression of SQSTM1, Beclin-1, LC3-II transition were analyzed by western blotting. β-actin was included as a loading control. **b** Working mechanism of tandem mRFP-GFP-LC3 adenovirus reporting system. **c** C2C12 myotubes over expressed mRFP-GFP-LC3 were treated with 10 μM NNC-55 or DMSO (control) for the indicated time and then subjected to confocal microscopy. The average numbers of green and red LC3 dots per cell in each condition were quantified, and over 30 cells were counted in each condition. **d** Ultrastructural features of myotubes treated with NNC-55 (10 μM) for 24 and 48 h were analyzed by electron microscopy. Typical images of the nucleus (N), endoplasmic reticulum (ER), and autophagosomes (yellow arrows) and autolysosome (green arrows) are shown at high magnification. These data are presented as the (mean ± SD) for three independent experiments. CON, control. *P < 0.05, **P < 0.01, ***P < 0.001. One-way analysis of variance (ANOVA).

Previous studies linked sustained ERS to the development of macroautophagy^40^. Beclin-1 is a key mediator of canonical macroautophagy development. NNC-55 did not induce the expression of Beclin-1 over the experimental time period. As this result did not support the induction of canonical autophagy in myotubes, we further analyzed the conversion of LC3 from its soluble form (LC3-I) to its autophagosome-associated form (LC3-II), which can be taken as an indirect measurement of the number of autophagosomes^41^. We found constitutive LC3-II conversion that was further increased in a time-dependent manner by treatments with NNC-55 (Fig. 4a).

The accumulation of LC3-II can be interpreted as indicating excessive autophagosome formation or reduced degradation. To clarify this, a flux experiment was performed with a tandem mRFP-GFP-LC3 adenovirus, a reporter used to conveniently monitor and quantify autophagy flux^42^. The mRFP signal was relatively stable compared with that of GFP, since functional fusion with the lysosome allows quenching of the acid-sensitive GFP signal (Fig. 4b). Treatment with NNC-55 produced many yellow puncta 24 h later, but no red puncta were observed even 48 h after treatment, reflecting distal autophagy blockade and lysosomal degradation impairment, producing persistent green and red fluorescence signals (Fig. 4c). We further analyzed the levels of SQSTM1, an adaptor protein that interacts with LC3 and is involved in the targeting of protein aggregates by autophagosomes. Consistent with the results regarding LC3-I to LC3-II conversion, a robust increase in the SQSTM1 protein band was observed in NNC-55-treated myotubes (Fig. 4a), indicating incomplete autophagy. Finally, TEM analysis revealed the acceleration of autophagosome formation, massive autolysosome accumulation and distal autophagy blockade (Fig. 4d).

### Further inhibition of autophagy promotes NNC-55-induced damage, while autophagy activation does not exert the opposite protective effect

**Fig. 5:**
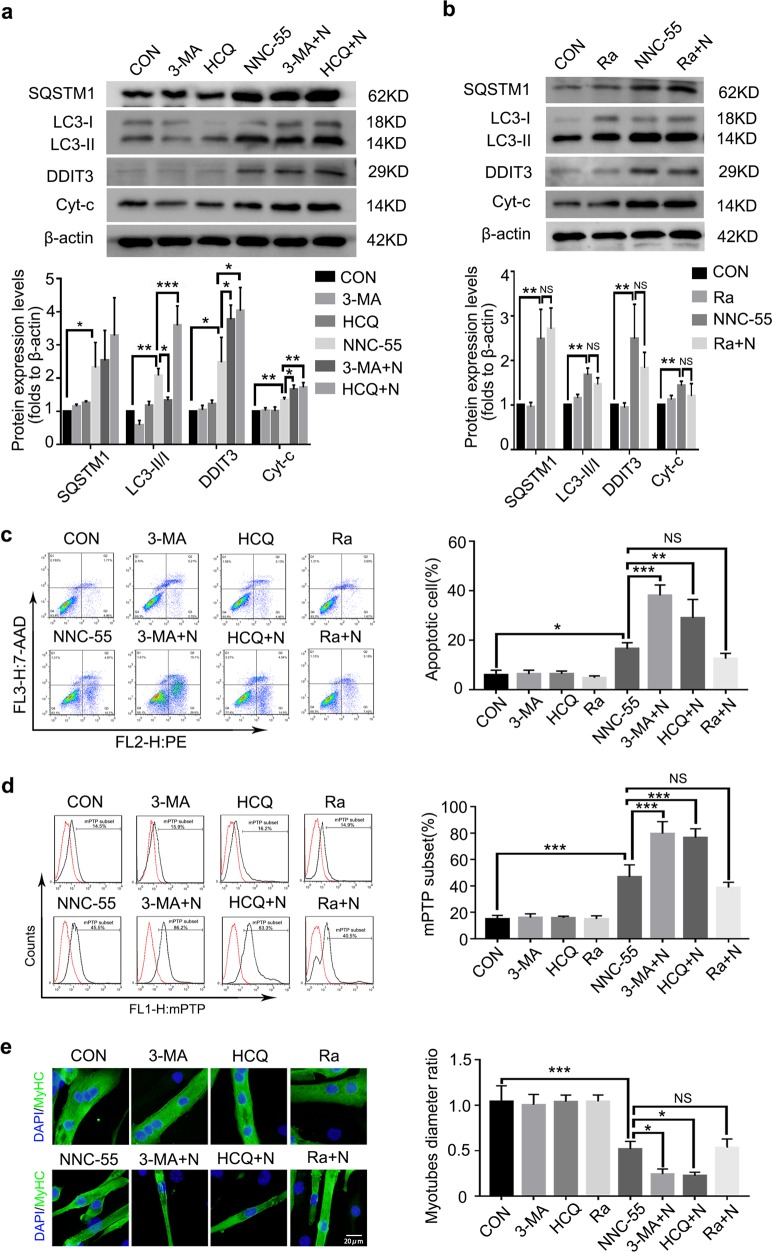
Further inhibition of autophagy enhances the damage induced by T-channel inhibitor, but autophagy activator does not rescue the damage. C2C12 myotubes were pre-treated with inhibitor (3-MA or HCQ) or activator (Ra) of autophagy for 1 h and then exposed to NNC-55 (10 μM) for another 24 h. **a**, **b** Western blotting analysis of SQSTM1, LC3, DDIT3 and Cyt-c expression levels in myotubes. β-actin was used as a loading control. **c**, **d** Apoptotic myotubes were detected with the annexin V-PE/7-AAD kit (**c**) and mitochondrial permeability were detected by mPTP kit (**d**) and then analyzed by flow cytometry. **e** The myotubes status were analyzed by anti-MyHC immunofluorescence staining. These data are presented as the (mean ± SD) for three independent experiments. CON, control. 3-MA + N, 3-MA + NNC-55. HCQ + N, HCQ + NNC-55. Ra + N, Rapamycin + NNC-55. NS, no significance. *P < 0.05, **P < 0.01, ***P < 0.001. One-way analysis of variance (ANOVA).

To further elucidate the role of autophagy in NNC-55-induced myotube atrophy and apoptosis, we analyzed the response of myotubes to the activation or inhibition of autophagy. 3-MA is a PI3K inhibitor widely used to inhibit autophagy via its inhibitory effect on class III PI3K. HCQ could block autophagy by impairing lysosomal function and causing the accumulation of autophagosomes with undigested contents. Treatment with 3-MA or HCQ alone did not affect myotube death or atrophy, but their combined application with NNC-55 significantly augmented DDIT3 and cytochrome c (Cyt-c) protein expression compared with that in the NNC-55-treated group (Figs. 5a, e). Moreover, HCQ further increased the accumulation of LC3-II induced by NNC-55, which also indicated the partial preservation of normal lysosomal function and complete autophagy after NNC-55 treatment in some cells.

Rapamycin is a potent and specific mTOR inhibitor and autophagy activator. We did not find a decrease in SQSTM1 or LC3-II conversion due to the inhibition of late autophagy after additional treatment with rapamycin. Meanwhile, no repression of apoptosis-related signals or myotube atrophy was found (Fig. 5b, e). Apoptosis and mPTP analysis also demonstrated that further autophagy inhibition promoted myotube injury and death while autophagy activator application had no effect (Fig. 5c, d).

### ERS is upstream of myotube atrophy, apoptosis and autophagy blockade induced by NNC-55

**Fig. 6:**
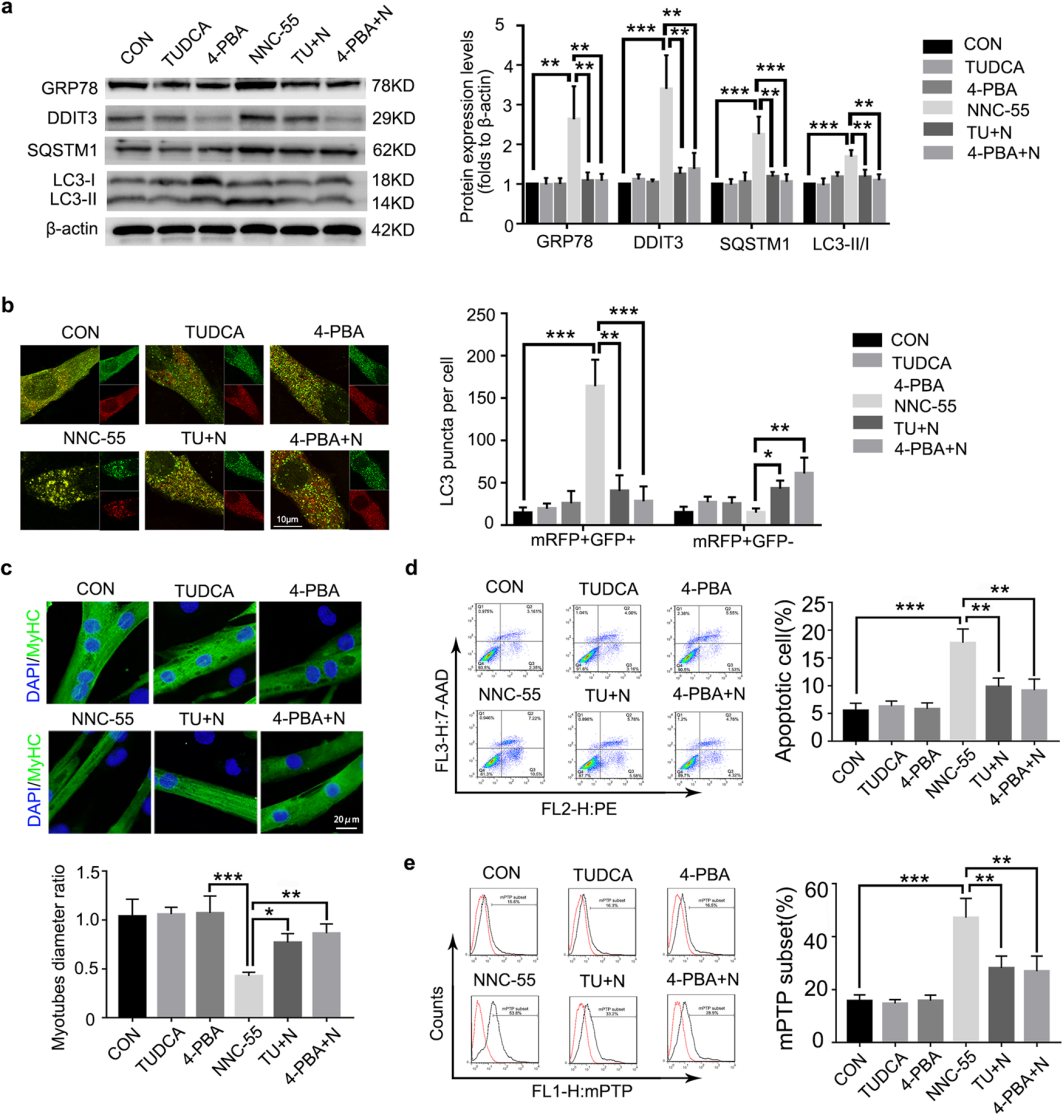
ERS inhibitors alleviate the damage and autophagy inhibition induced by T-channel inhibitor. C2C12 myotubes were pre-treated with ER stress inhibitors (4-PBA or TUDCA) for 1 h and then exposed to NNC-55 (10 μM) for another 24 h. **a** Western blotting analysis of GRP78, DDIT3, SQSTM1 and LC3 expression levels in myotubes. β-actin was used as a loading control. **b** Myotubes over expressed mRFP-GFP-LC3 were treated with 10 μM NNC-55 in combination with 4-PBA or TUDCA, and the cells with mRFP-GFP-LC3 punctate dots were examined by confocal microscope. **c** The myotubes status were analyzed by anti-MyHC immunofluorescence staining. **d**, **e** Apoptotic myotubes were detected with the annexin V-PE/7-AAD kit (**d**) and mitochondrial permeability were detected by mPTP kit (**e**) and then analyzed by flow cytometry. These data are presented as the (mean ± SD) for three independent experiments. CON, control. TU + N, TUDCA + NNC-55. 4-PBA + N, 4-PBA + NNC-55. *P < 0.05, **P < 0.01, ***P < 0.001. One-way analysis of variance (ANOVA).

Studies have shown that ERS can induce changes in autophagy^29,40^. However, we could not infer the regulatory relationship between ERS and autophagy, because the inhibition of both ERS and autophagy was most pronounced after 24 h of NNC-55 treatment (Figs. 2b and 3a). We further investigated the regulatory effect of ERS on autophagy. 4-PBA is an inhibitor of ERS that inhibits the UPR to reduce ER pressure. TUDCA, a classic ERS suppressor, stabilizes the ER structure to reduce ERS. Our study revealed that 4-PBA and TUDCA in combination with NNC-55 significantly inhibited expression of the apoptosis-related proteins DDIT3 and Cyt-c and reduced the accumulation of SQSTM1 and LC3-II compared with that in the NNC-55-treated group (Fig. 6a). This result indicated that ERS could be an upstream signal that inhibits autophagy flux in NNC-55-treated myotubes. The formation of LC3 puncta was reduced after ERS was relieved, indicating that autophagy was inhibited in the early stage or that its blockade was alleviated in the terminal stage (Fig. 6b). Combined with studies on myotube diameter, apoptosis and mitochondrial membrane permeability, we observed the protective effect of ERS inhibitors on myotube atrophy, injury and apoptosis induced by NNC-55 (Fig. 6c–e). At the same time, we could also infer that ERS inhibitors were more likely to protect myotubes by alleviating the inhibition of autophagy at the late stage but not inhibiting early autophagy induction.

### Gene silencing of CACNA1H mimics the effects of NNC-55 on myotube atrophy and apoptosis induction

**Fig. 7:**
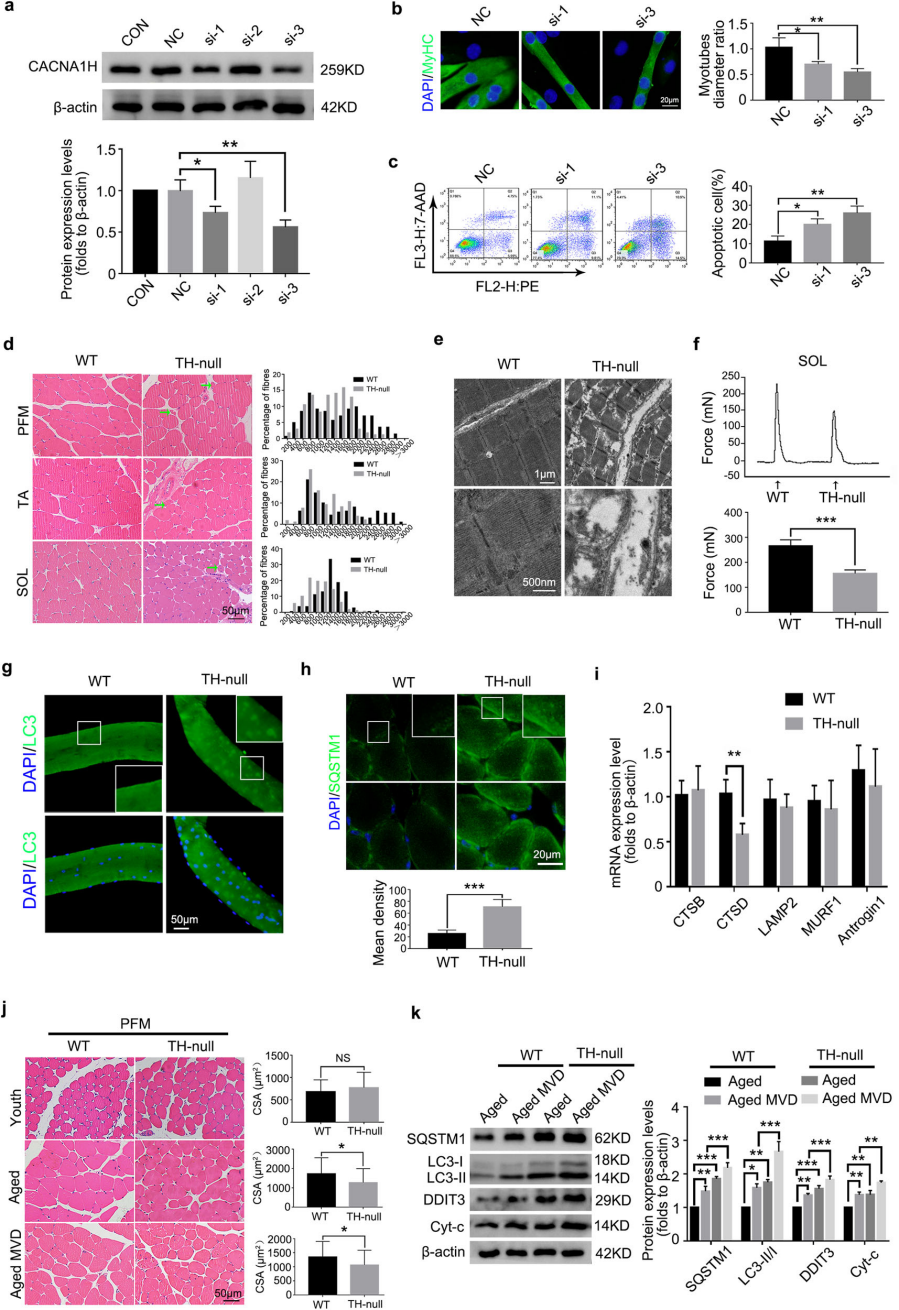
CACNA1H gene silence promotes myotubes atrophy and apoptosis and TH-null mice exhibit impaired muscle function. **a**–**c** To observe the function of CACNA1H, C2C12 myotubes were treated with the CACNA1H siRNA and the effectiveness was verified by western blotting (**a**). The myotubes status were analyzed by anti-MyHC immunofluorescence staining (**b**) and apoptotic myotubes were detected with the annexin V-PE/7-AAD kit and then analyzed by flow cytometry (**c**). **d**–**i** 4-month-old female WT and TH-null mice were used for muscle feature analysis (*n* = 7). The distribution of CSA of the PFM, TA and SOL was evaluated by HE staining (**d**). Ultrastructural features of PFM were analyzed by electron microscopy (**e**). The isometric twitch force of SOL was measured through the tension transducer and PowerLab data acquisition system (**f**). Single myofibers were extracted from the extensor digitorum longus (EDL) muscle and then fixed with formaldehyde for LC3 immunostaining (**g**). The expression of SQSTM1 in PFM was evaluated by immunofluorescence (**h**). CTSB, CTSD, LAMP2, MURF1 and Antrogin1 gene expression in PFM were detected by qRT-PCR (**i**). **j**, **k** The PFM of the youth (Youth, 1-month old), aged virgin (Aged, 12-momth old) and aged multiple vaginal-delivery (Aged MVD, 12-momth old) group, WT and TH-null mice (*n* = 7) were used for CSA analysis by HE staining (**j**), and SQSTM1, LC3, DDIT3 and Cyt-c protein expression measurement by western blotting (**k**). These data are presented as the (mean ± SD). CON, control. NC, negative control. Aged MVD, aged multiple vaginal-delivery. PFM, pelvic floor muscle. TA, anterior tibial muscle. SOL, soleus muscle. *P < 0.05, **P < 0.01, ***P < 0.001. **a**–**c**, **f**, **h**, **i**, **j** Unpaired two-tailed Student’s t-test. **k** One-way analysis of variance (ANOVA).

To genetically analyze the role of the T-channel, we measured myotube apoptosis and diameter with or without transfection with siRNA targeting CACNA1H, the most substantially expressed T-channel subtype in skeletal muscle (Fig. 1). The knockdown efficiency was determined using western blotting, which demonstrated a 30% reduction in CACNA1H protein expression under treatment with si-1 and a 50% reduction under treatment with si-3 (Fig. 7a). MyHC immunofluorescence staining showed a reduced myotube diameter after siRNA treatment (Fig. 7b). Flow cytometry analysis indicated increased myotube apoptosis when CACNA1H expression was decreased (Fig. 7c). These studies indicate that the dysfunction of CACNA1H plays an important role in myotube atrophy and apoptosis.

### Ablation of CACNA1H leads to skeletal muscle damage, atrophy and lysosome-associated autophagy inhibition in vivo

To understand the role of CACNA1H in the regulation of skeletal muscle function, we obtained the PFM, TA and SOL tissues of 4-month-old female WT and Cacna1h^−/−^ (TH-null) mice and compared the distribution of their myofiber sizes. The results showed that TH-null muscles had an increased number of smaller myofibers. The CSA of the PFM, TA and SOL of TH-null mice mainly ranged from 600 to 1800, 800 to 1800 and 600 to 1400 μm^2^, respectively (Fig. 7d). Ultrastructural analysis of the PFM showed that the sarcomeres of TH-null mice were not shorter than those of WT mice, but while when had been knocked out, the SR in the PFM of TH-null mice was expanded, myofibers had partially dissolved and visible mitochondria had expanded and dissolved (Fig. 7e). The maximal isometric twitch force of the SOL of TH-null mice was significantly lower than that of WT mice, indicating impaired skeletal muscle function (Fig. 7f).

Autophagy levels are directly reflected by the number of punctate LC3^41^. Punctuate LC3 aggregation was detected in TH-null mice by single myofiber culture and immunohistochemical staining analysis (Fig. 7g). At the same time, we also found the accumulation of SQSTM1 in the PFM of TH-null mice (Fig. 7h). A gene study showed that the lysosomal cathepsin D (CTSD) transcript was decreased when TH was silenced (Fig. 7i), whereas the expression of LAMP-2, a receptor at the lysosomal membrane crucial for the completion of the lysosomal-autophagic degradation, did not change, suggesting that autolysosome accumulation results from reduced degradation. Results of qRT-PCR also serve as evidence of the unchanged levels of mRNAs coding for atrogin-1 and MURF1, important ubiquitin protein E3 ligases in muscular atrophy, further supporting the importance of ERS and autophagy blockade in CACNA1H deficiency-induced muscle atrophy.

### MVD-associated decline in CACNA1H expression promotes PFM atrophy and PFD

**Fig. 8:**
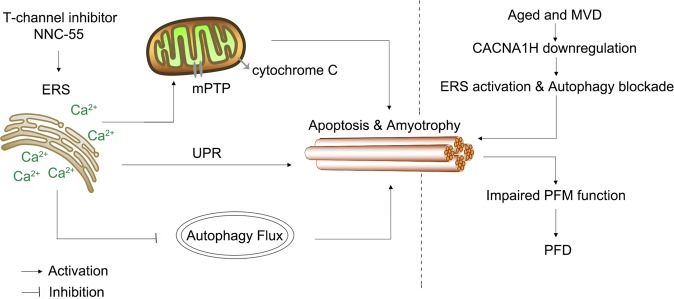
Schematic diagram depicts how CACNA1H downregulation induces PFD. The expression of CACNA1H reduces in PFM of elderly MVD mice. The inhibition of T-channel induces ERS and autophagy flux inhibition and ultimately results in myofiber atrophy and apoptosis, which is the possible mechanism for PFM function impairment and PFD induced by CACNA1H downregulation. ERS, endoplasmic reticulum stress. UPR, unfolding protein response. MVD, multiple vaginal delivery. PFM, pelvic floor muscle. PFD, pelvic floor dysfunction.

Finally, we detected the CSA of the PFM of young mice (1-month old), old virgin mice and old mice after MVD and found no differences in the CSA of the PFM in the young mice, but a significant difference in the old virgin mice and old mice after MVD, and MVD further reduced the average CSA of the PFM (Fig. 7j). These results showed for the first time that CACNA1H was more likely to function by promoting muscular atrophy than by generating congenital muscle. Meanwhile, MVD and CACNA1H downregulation led to an increase in the expression of SQSTM1 and LC3-II as well as expression of the apoptosis-related factors DDIT3 and Cyt-c in old mice (Fig. 7k). This also demonstrated that downregulation of CACNA1H could inhibit autophagy flux and promote apoptosis. Collectively, these results showed that functional decline of CACNA1H led to ERS and inhibited autophagy flux to induce myofiber atrophy and apoptosis, which ultimately resulted in PFM atrophy and PFD (Fig. 8).

## Discussion

Pelvic organ prolapse, including vaginal wall prolapse, uterine prolapse and rectal prolapse, is one of the most common manifestations of PFD in women. Its occurrence is related to a decline in the function of PFM^6^. As the PFM is the key muscular structure in the pelvis, mechanical support provided by the PFM is the main factor that maintains the correct positions and functions of organs in the pelvic area^43–45^. Pregnancy, MVD and age are important factors that damage the PFM^5,7,46^. In skeletal muscle, CACNA1H is the major subunit for T-channels (Fig. 1a, b). A shown by the pathogenesis, we first found that the expression of CACNA1H in the PFM of old mice after MVD was downregulated compared to that in old mice, indicating that CACNA1H may have a regulatory effect on the generation of PFD.

Blockade of T-channel function interfered with Ca^2+^ signaling in the early stage of myoblast differentiation and inhibited differentiation in the fusion stage^13^. However, it is worth emphasizing that we did not find congenital muscular atrophy or obvious myopathies in the PFM of 1-month-old TH-null mice (Fig. 7j and Supplementary Fig. 1b), which indicates that CACNA1H is not necessary in muscle development, or there is a possible compensatory effect in the organism during muscle development. In this study, we ruled out the possibility that CACNA1G expression increased as a compensation for CACNA1H knockout (Supplementary Fig. 1c). Meanwhile, we found that compared with 1-month-old TH-null mice, 12-month-old mice had more obvious muscular atrophy of the PFM, and MVD exacerbated this damage (Fig. 7j). In adult mice, muscular atrophy was more pronounced in the TA (Fig. 7d), where fast glycolytic type IIb fibers were more dominant than in the PFM, which mainly contains slow oxidative type I and fast oxidative type Iiα fibers^47^. This suggests that the effect of the loss of CACNA1H function on skeletal muscle atrophy becomes more pronounced over time and with accumulating microdamage.

Skeletal muscle undergoes continuous remodeling to survive in response to physiological, mechanical and metabolic stress. Regulation of proteostasis by the ER plays a crucial physiological role in the maintenance of skeletal muscle and sarcomeres in response to various stresses. Our study revealed that a T-channel inhibitor could lead to abnormal Ca^2+^ accumulation in the cytoplasm, ERS and apoptosis (Fig. 2), which may be related ERS induced by the continuous disturbance of Ca^2+^ or the release of intracellular Ca^2+^ from ER stores induced by ERS and subsequent Cyt-c release from mitochondria^48^. In addition to ERS, our study also found that T-channel inhibitor intervention inhibited autophagy flux, which is consistent with previous research^31,33,49^. With ERS inhibitor analysis, we demonstrate ERS is an upstream signal and could be used as a therapeutic target for CACNA1H downregulation-induced muscular atrophy. The inhibition of autophagy flux might be attributed to the reduced digestive ability of lysosomes, which would explain why the autophagy activator rapamycin did not have a protective effect.

In summary, our results highlight the role of CACNA1H as a key regulator in the maintenance of complex regulatory networks in muscle. CACNA1H-deficient muscles exhibited a reduced capacity, and this decreased contractile force is likely attributed to the disturbance of Ca^2+^ regulation and ERS. The decreased CACNA1H expression in the PFM of old mice after MVD and the force decline in CACNA1H-deficient muscle revealed that the reduced expression of CACNA1H related to MVD is associated with muscular atrophy and PFD. Together with the results of in vitro study, our current study underlines the importance of CACNA1H in skeletal muscle function maintenance.

## Supplementary information

Supplementary Figure 1

Supplementary Figure 1 legend

Supplementary tables

## References

[1] Zhu L (2009). The epidemiological study of women with urinary incontinence and risk factors for stress urinary incontinence in China. Menopause.

[2] Chong EC, Khan AA, Anger JT (2011). The financial burden of stress urinary incontinence among women in the United States. Curr. Urol. Rep..

[3] Wilson L, Brown JS, Shin GP, Luc KO, Subak LL (2001). Annual direct cost of urinary incontinence. Obstet. Gynecol..

[4] Wu JM (2014). Prevalence and trends of symptomatic pelvic floor disorders in U.S. women. Obstet. Gynecol..

[5] Blomquist JL, Munoz A, Carroll M, Handa VL (2018). Association of Delivery Mode With Pelvic Floor Disorders After Childbirth. JAMA.

[6] Alperin M, Cook M, Tuttle LJ, Esparza MC, Lieber RL (2016). Impact of vaginal parity and aging on the architectural design of pelvic floor muscles. Am. J. Obstet. Gynecol..

[7] Cook MS, Bou-Malham L, Esparza MC, Alperin M (2017). Age-related alterations in female obturator internus muscle. Int. Urogynecol..

[8] Bernhardt ML (2015). CaV3.2 T-type channels mediate Ca2+ entry during oocyte maturation and following fertilization. J. Cell Sci..

[9] Vastagh C, Solymosi N, Farkas I, Liposits Z (2019). Proestrus Differentially Regulates Expression of Ion Channel and Calcium Homeostasis Genes in GnRH Neurons of Mice. Front. Mol. Neurosci..

[10] Jimenez H (2019). Tumour-specific amplitude-modulated radiofrequency electromagnetic fields induce differentiation of hepatocellular carcinoma via targeting Cav3.2 T-type voltage-gated calcium channels and Ca2+ influx. EBioMedicine.

[11] Cain SM (2018). CaV 3.2 drives sustained burst-firing, which is critical for absence seizure propagation in reticular thalamic neurons. Epilepsia.

[12] Berthier C, Monteil A, Lory P, Strube C (2002). Alpha(1H) mRNA in single skeletal muscle fibres accounts for T-type calcium current transient expression during fetal development in mice. J. Physiol..

[13] Bijlenga P (2000). T-type alpha 1H Ca2+ channels are involved in Ca2+ signaling during terminal differentiation (fusion) of human myoblasts. Proc. Natl Acad. Sci. USA.

[14] Luin E, Ruzzier F (2007). The role of L- and T-type Ca2+ currents during the in vitro aging of murine myogenic (i28) cells in culture. Cell Calcium.

[15] Carter MT, Mcmillan HJ, Tomin A, Weiss N (2019). Compound heterozygous CACNA1H mutations associated with severe congenital amyotrophy. Channels.

[16] Agrawal A, Suryakumar G, Rathor R (2018). Role of defective Ca^2+^ signaling in skeletal muscle weakness: Pharmacological implications. J. Cell Commun. Signal..

[17] Verfaillie T (2012). PERK is required at the ER-mitochondrial contact sites to convey apoptosis after ROS-based ER stress. Cell Death Differ..

[18] Welihinda AA, Tirasophon W, Kaufman RJ (1999). The cellular response to protein misfolding in the endoplasmic reticulum. Gene Expr..

[19] Karagoz GE, Aragon T, Acosta-Alvear D (2019). Recent advances in signal integration mechanisms in the unfolded protein response. F1000Res.

[20] Yoshida H, Matsui T, Yamamoto A, Okada T, Mori K (2001). XBP1 mRNA is induced by ATF6 and spliced by IRE1 in response to ER stress to produce a highly active transcription factor. Cell.

[21] Kim I, Xu W, Reed JC (2008). Cell death and endoplasmic reticulum stress: disease relevance and therapeutic opportunities. Nat. Rev. Drug Discov..

[22] Bohnert KR, McMillan JD, Kumar A (2018). Emerging roles of ER stress and unfolded protein response pathways in skeletal muscle health and disease. J. Cell Physiol..

[23] Moorwood C, Barton ER (2014). Caspase-12 ablation preserves muscle function in the mdx mouse. Hum. Mol. Genet..

[24] Ikezoe K (2007). Endoplasmic reticulum stress in myotonic dystrophy type 1 muscle. Acta Neuropathol..

[25] Rabinowitz JD, White E (2010). Autophagy and metabolism. Science.

[26] Ouyang L (2012). Programmed cell death pathways in cancer: a review of apoptosis, autophagy and programmed necrosis. Cell Prolif..

[27] Jiao J, Demontis F (2017). Skeletal muscle autophagy and its role in sarcopenia and organismal aging. Curr. Opin. Pharmacol..

[28] Garcia-Prat L (2016). Autophagy maintains stemness by preventing senescence. Nature.

[29] Yu Z (2011). Macroautophagy is regulated by the UPR-mediator CHOP and accentuates the phenotype of SBMA mice. PLoS Genet..

[30] Sano R (2012). Endoplasmic reticulum protein BI-1 regulates Ca2+-mediated bioenergetics to promote autophagy. Genes Dev..

[31] Das A (2013). T-type calcium channel blockers inhibit autophagy and promote apoptosis of malignant melanoma cells. Pigment Cell Melanoma Res..

[32] Visa A (2019). T-Type Cav3.1 channels mediate progression and chemotherapeutic resistance in glioblastoma. Cancer Res..

[33] Niklasson M (2017). Membrane-depolarizing channel blockers induce selective glioma cell death by impairing nutrient transport and unfolded protein/amino acid responses. Cancer Res..

[34] Yi, Y. et al. Effects of mechanical trauma on the differentiation and ArfGAP3 expression of C2C12 myoblast and mouse levator ani muscle. Preprint at 10.1007/s00192-019-04212-4 (2020). 10.1007/s00192-019-04212-4 31989201

[35] Tang J (2019). Protective role of nuclear factor erythroid-2-related factor 2 against mechanical trauma-induced apoptosis in a vaginal distension-induced stress urinary incontinence mouse model. Oxid. Med. Cell Longev..

[36] Li S (2019). Electrical stimulation activates fibroblasts through the elevation of intracellular free ca2+: potential mechanism of pelvic electrical stimulation therapy. Biomed. Res. Int..

[37] Huang L (2004). NNC 55-0396 [(1S,2S)-2-(2-(N-[(3-benzimidazol-2-yl) propy l]-N-methylamino)ethyl)-6-fluoro-1,2,3,4-tetrahydro-1-isopropyl-2-naphtyl cyclopropanecarboxylate dihydrochloride]: a new selective inhibitor of T-type calcium channels. J. Pharmacol. Exp. Ther..

[38] Brown GN, Leong PL, Guo XE (2016). T-Type voltage-sensitive calcium channels mediate mechanically-induced intracellular calcium oscillations in osteocytes by regulating endoplasmic reticulum calcium dynamics. Bone.

[39] Chen D, Wang Y, Chin ER (2015). Activation of the endoplasmic reticulum stress response in skeletal muscle of G93A*SOD1 amyotrophic lateral sclerosis mice. Front Cell Neurosci..

[40] Bhardwaj M., Leli N. M. & Koumenis C, Amaravadi R K, Regulation of autophagy by canonical and non-canonical ER stress responses. Preprint at 10.1016/j.semcancer.2019.11.007 (2019). 10.1016/j.semcancer.2019.11.007 PMC7325862 31838023

[41] Lazova R (2012). Punctate LC3B expression is a common feature of solid tumors and associated with proliferation, metastasis, and poor outcome. Clin. Cancer Res..

[42] Gao L (2019). Paeonol induces cytoprotective autophagy via blocking the Akt/mTOR pathway in ovarian cancer cells. Cell Death Dis..

[43] DeLancey JO (1997). The pathophysiology of stress urinary incontinence in women and its implications for surgical treatment. World J. Urol..

[44] DeLancey JO (1994). Structural support of the urethra as it relates to stress urinary incontinence: the hammock hypothesis. Am. J. Obstet. Gynecol..

[45] DeLancey JO (2016). What’s new in the functional anatomy of pelvic organ prolapse?. Curr. Opin. Obstet. Gynecol..

[46] Van Geelen H, Ostergard D, Sand P (2018). A review of the impact of pregnancy and childbirth on pelvic floor function as assessed by objective measurement techniques. Int. Urogynecol. J..

[47] Helt M, Benson JT, Russell B, Brubaker L (1996). Levator ani muscle in women with genitourinary prolapse: indirect assessment by muscle histopathology. Neurourol. Urodyn..

[48] Deniaud A (2008). Endoplasmic reticulum stress induces calcium-dependent permeability transition, mitochondrial outer membrane permeabilization and apoptosis. Oncogene.

[49] Pushparaj C (2015). Voltage-gated calcium channel blockers deregulate macroautophagy in cardiomyocytes. Int. J. Biochem. Cell Biol..

