# Supplementary figures and images for: CACNA1H downregulation induces skeletal muscle atrophy involving endoplasmic reticulum stress activation and autophagy flux blockade
Source: Cell Death Dis. 2020 Apr 24;11(4):279. doi: 10.1038/s41419-020-2484-2 (PMC7181873; doi:10.1038/s41419-020-2484-2)

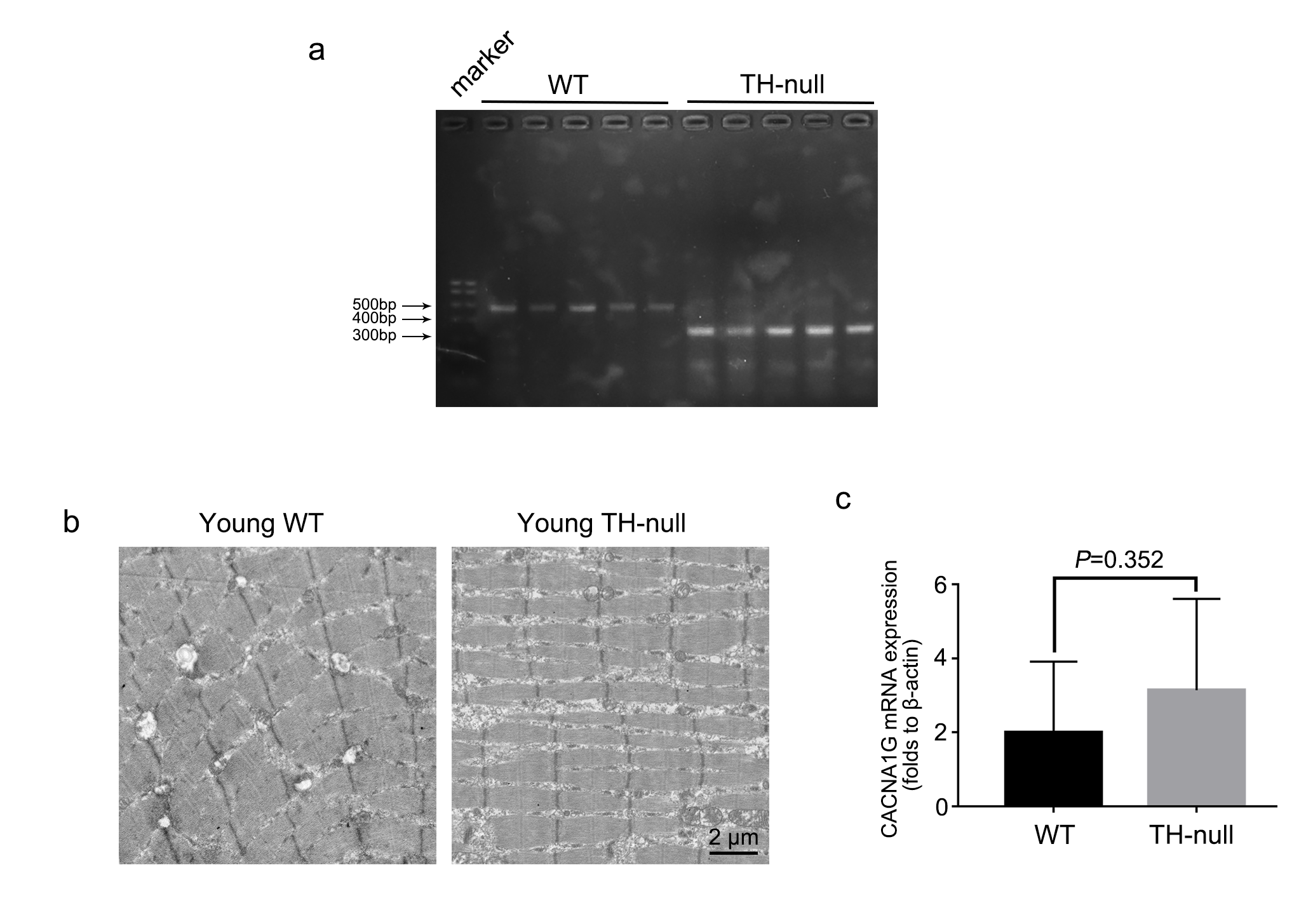

Supplement: Supplementary file 1 — Supplementary Figure 1 [file 41419_2020_2484_MOESM1_ESM.tif]
