# Supplementary material for: CACNA1H downregulation induces skeletal muscle atrophy involving endoplasmic reticulum stress activation and autophagy flux blockade
Source: Cell Death Dis. 2020 Apr 24;11(4):279. doi: 10.1038/s41419-020-2484-2 (PMC7181873; doi:10.1038/s41419-020-2484-2)
Supplement: Supplementary file 2 — Supplementary Figure 1 legend [file 41419_2020_2484_MOESM2_ESM.docx]

**Supplementary Figure 1 legend:** **a.** Identification of TH-null mice (Cacna1h-/-), Mutant (TH-null) = 330 bp; Wild type (WT) = 480 bp. **b.** Ultrastructural features of PFM in 1-month-old mice were analyzed by electron microscopy (n=5). **c.** CACNA1G mRNA expression in 1-month-old WT and TH-null mice was detected by qRT-PCR (n=7). Data are presented as the (mean ± SD). **c.** Unpaired two-tailed Student’s t-test.
