# Supplementary material for: CACNA1H downregulation induces skeletal muscle atrophy involving endoplasmic reticulum stress activation and autophagy flux blockade
Source: Cell Death Dis. 2020 Apr 24;11(4):279. doi: 10.1038/s41419-020-2484-2 (PMC7181873; doi:10.1038/s41419-020-2484-2)
Supplement: Supplementary file 3 — Supplementary tables [file 41419_2020_2484_MOESM3_ESM.docx]

**Supplementary Information**

**Supplementary Table 1: Primers for Cacna1h-/- (TH-null) mice identification**

| **Primer name** | **Specific sequence (5’----3’)** |
| --- | --- |
| PRIMER-11395 | ATT CAA GGG CTT CCA CAG GGT A |
| PRIMER-11396 | CAT CTC AGG GCC TCT GGA CCA C |
| oIMR2063 | GCT AAA GCG CAT GCT CCA GAC TG |

**Supplementary Table 2：Primers for qRT-PCR**

| **Gene** | **Specific sequence (5’----3’)** |
| --- | --- |
| **CACNA1G** | FORWARD：GTCAGGAGAGCCAGGATGAG |
|  | REVERSE：TCAGGCAGGTCAAAGGAACT |
| **CACNA1H** | FORWARD：GCAGCCATCCTCGTCAATAC |
|  | REVERSE：AGCATCTCCAAGGCAAACAT |
| **ATF6** | FORWARD：TTCCTCCAGTTGCTCCATCTCCTC |
|  | REVERSE：AGGACCAGTGACAGGCTTCTCTTC |
| **IRE1** | FORWARD：TTGCCTCTTCAGATTCTGAGTC |
|  | REVERSE：GGGGAAGGACATTTGAAAAACA |
| **PERK** | FORWARD：GAGATCTGGCTCAAAGACGAAA |
|  | REVERSE：AAGGAGCTATGACTTCGATCTG |
| **CTSB** | FORWARD：CTCATGTAGGCTGCTTACCATA |
|  | REVERSE：TCTCCTTCACACTGTTAGACAC |
| **CTSD** | FORWARD：CATCTATCCGTCGGACTATGAC |
|  | REVERSE：ATCAAAGACGACTGTGAAACAC |
| **LAMP2** | FORWARD：GTGTGTGAAGAAGACCAAACTC |
|  | REVERSE：CTAATGCTGTAGTTTCCAACGG |
| **MuRF1** | FORWARD：ATCACGCAGGAGCAGGAGGAG |
|  | REVERSE：CTTGGCACTTGAGAGGAAGGTAGC |
| **Atrogin-1** | FORWARD：TTCACAAAGGAAGTACGAAGGA |
|  | REVERSE：GCTGGTCTTCAAGAACTTTCAG |
| **ATCB** | FORWARD: CTACCTCATGAAGATCCTGAC |
|  | REVERSE: CACAGCTTCTCTTTGATGTCAC |

**Supplementary Table 3：Core sequence of siRNA for CACNA1H**

| **Name** | **Specific sequence (5’----3’)** |
| --- | --- |
| si-m-Cacna1h_001 (si-1) | GGGTAAACATCATGTACGA |
| si-m-Cacna1h_002 (si-2) | GGAATGTGGTTCTTTACAA |
| si-m-Cacna1h_003 (si-3) | GTCGCATTGTAGACAGCAA |
